# Modified acid-PAGE method for rapid screening and phenotyping of wheat gliadin mutant lines

**DOI:** 10.1016/j.mex.2020.100858

**Published:** 2020-03-20

**Authors:** Hannah Watry, Alexander Zerkle, Debbie Laudencia-Chingcuanco

**Affiliations:** a USDA-ARS, Western Regional Research Center, 800 Buchanan Street, Albany, CA 94710, U.S.A.

**Keywords:** A-PAGE, Wheat, Gliadin, Mutant, Acid-PAGE, Gluten, A-PAGE, acidic-polyacrylamide gel electrophoresis, SDS-PAGE, sodium dodecyl sulfate polyacrylamide gel electrophoresis, HMW-GS, high-molecular glutenin subunit, LMW-GS, low-molecular weight glutenin subunit, APS, ammonium persulfate, TEMED, *N,N,N’,N’*-tetramethyl-ethylenediamine

## Abstract

Acid-polyacrylamide gel electrophoresis (A-PAGE) is used to phenotype different varieties of wheat based on their gliadin profiles. The family of gliadin proteins is a major component of wheat gluten. Gluten is the major determinant of the unique viscoelastic property of wheat dough that is necessary in the production of important food products including bread, cake, cookies and pasta. However, several gliadin proteins are also known to be causal agents in triggering human immunogenic responses that lead to several gluten-related health risks like celiac disease and wheat-dependent exercise-induced anaphylaxis. Therefore, research to identify wheat lines with reduced levels of immunogenic proteins is being vigorously pursued in several laboratories around the world. Unfortunately, no commercial A-PAGE gels are currently available for cereal researchers to use for separating wheat gliadins. This work reports the development of an easy-to-use A-PAGE protocol to resolve gliadins with high reproducibility and resolution to screen and phenotype gliadin deficient lines in wheat.

• This acetic acid based A-PAGE method with urea utilizes commercially available reagents and materials to make gel casting simpler and more efficient.
• It can be used to phenotype different wheat varieties to establish purity and to identify mutants of wheat with altered gliadin protein profiles.

## Graphical abstract

**Figure:**
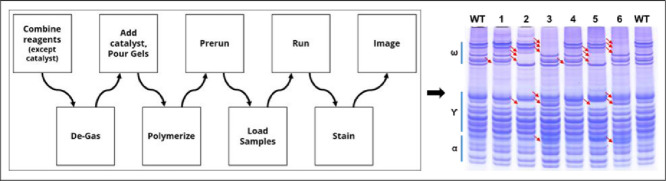

**Table:** Specifications Table

Subject Area:	Agricultural and Biological Sciences
More specific subject area:	*Crop Improvement*
Method name:	*Acid-PAGE*
Name and reference of original method:	Porter E., Valore E.V., Anouseyan R., Salzman N.H., Detection of antimicrobial (poly)peptides with acid urea polyacrylamide gel electrophoresis followed by western immunoblot, Methods Mol. Biol. 1225 (2015) 105115.

## Introduction

### Method details

This method modified a previously published protocol [1] on the preparation of A-PAGE gels used to purify and characterize anti-bacterial peptides and adapted it to separate gliadins in wheat. Changes were made to the reagent composition for gel casting in order to make gels compatible with wheat gliadins. The protocol was also optimized for faster polymerization, firmer gel constituency, and shorter run time, as well as the use of commercially available reagents and equipment. Instead of using customized assembled glass plates, commercially available empty gel cassettes were used for casting, which eliminated reagent leakage during polymerization, improved gel uniformity and saved time. The time it takes to run the gel to completion was shortened to almost half (from > 180 minutes to 90–100 min) compared to the lactate-lactic acid based method. The gliadin bands resolved from different wheat genotypes are distinct and consistent between runs, indicating that our method is a reliable way to phenotype different wheat varieties to assess purity in a breeding program. The method is easy to perform and faster than previous protocols, and therefore can facilitate the screening for altered gliadin protein profiles of variants from a mutagenized population.

### Background

Gluten is the major determinant of wheat dough's unique visco-elastic property that is critical in the production of food products like bread, cookies, cake and pasta. The gluten is composed mainly of two types of seed storage proteins: the glutenins and gliadins. Both the glutenin and gliadin proteins are encoded by multi-gene families. Glutenins are categorized further into high- and low-molecular weight subunits (HMW-GS and LMW-GS, respectively) based on their mobility on an SDS-PAGE gel [2, 3]. Gliadins, on the other hand, are classified further into omega-, gamma- and alpha-gliadins based on their mobilities on an A-PAGE gel [4]. In hexaploid wheat, HMW-GS and LMW-GS genes are encoded by the *Glu1* and *Glu3* loci that map to the chromosome 1 group long and short arm, respectively [5]. The omega- and gamma-gliadins are encoded by the *Gli1* loci that are tightly linked to LMW-GS *Glu3* loci in the short arms of the chromosome 1 group. The alpha-gliadins are encoded by *Gli2* loci in the short arms of chromosome 6 group [6,7]. The different alleles of each member of these gluten gene families contribute to the variability of wheat dough performance which is harnessed to create different food products.

Several human health-risks are associated with the consumption of wheat-derived food [8]. Components of gluten have been shown to be the causative agents in triggering a majority of these immunogenic health risks which include celiac disease (CD) and wheat-dependent exercise-induced anaphylaxis (WDEIA). The gliadins have been shown to contain a significant number of epitopes that trigger these gluten-related disorders [9], [10], [11], [12]. The search for and development of wheat lines with reduced immunogenic potential is an area of research that has gained more attention in recent years [13], [14], [15], [16]. Although biotechnological approaches like the use of RNAi and CRISPR/Cas9 have been successfully used to reduce targeted gliadins in wheat, the lengthy regulatory approval time needed and the current negative customer perception of food products derived through biotechnological approaches has hampered the utilization of these new germplasm. Thus, conventional breeding approaches using natural variants or with induced mutations using traditional mutagens e.g. chemical and radiation, are still being used to develop wheat lines with reduced immunogenic gluten proteins.

The use of polyacrylamide gel electrophoresis is an essential tool in the search and characterization of novel wheat lines with reduced immunogenic potential. We used commercially available reagents and pre-cast SDS-PAGE gels to screen a fast-neutron radiation mutagenized population of a commercial bread wheat variety to identify lines with glutenin deficiencies [17,18]. Standardized SDS-PAGE reagents and pre-cast gels have been commercially available for years, but A-PAGE gels and reagents are not. We needed a consistent and efficient method to screen the same large mutagenized population to identify novel lines with deficiencies in gliadins. To address this issue, we developed this A-PAGE protocol.

## Materials and methods

### Reagents and equipment

SureCast™ 40% (w/v) Acrylamide 29:1 acrylamide:bis-acrylamide, 450 mL, (Thermo Fisher Scientific, catalog no. HC2040)

SureCast™ TEMED (Thermo Fisher Scientific, catalog no. HC2006)

Urea, Powder (J.T. Baker, catalog no. 4111-05)

Acetic Acid (17.4 M) (Fisher Chemical, catalog no. A38S)

Ammonium Persulfate (Thermo Fisher Scientific, catalog no. 17874)

1 M Tris pH 8.5

Resin Beads (Bio-Rad AG® 501-X8 Mixed Bed Resin, biotechnology grade, 100 g #1437424)

Coomassie Brilliant Blue R (Sigma-Aldrich, catalog no. B-0149)

Phosphoric acid >85% (Sigma-Aldrich, catalog no. 438081-500 ml)

Ammonium sulfate (Fisher Scientific, catalog no. A702-500)

Methanol (Fisher Scientific, catalog no. A412-4)

Deionized Water (e.g. Milli-Q® water)

Methyl Green Tracking Dye (Sigma-Aldrich, catalog no. M8884)

Empty Gel Cassettes, mini, 1.0 mm, 25 pack (Thermo Fisher Scientific, catalog no. NC2010)

Empty Gel Cassette Combs, mini, 1.0 mm 10-well combs, 25 pack (Thermo Fisher Scientific, catalog no. NC3010)

XCell SureLock™ Mini-Cell (Thermo Fisher Scientific, catalog no. EI0001)

Buffer Dam for XCell SureLock™ (Thermo Fisher Scientific, catalog no. EI0012)

Electrophoresis Power Supply (e.g. Thermo Fisher Scientific EC1000XL)

Gel staining box (e.g. Thermo Scientific™ Owl™ Gel Staining Box, catalog no. OW-GSB-3)

Thermocontrolled Incubator (e.g. VWR Scientific 1525 Incubator)

Microcentrifuge

Vortexer

Micropipettes

125 mL Pyrex flask with side-arm

In-house vacuum source

Protein gel imager, camera, or document scanner

### Biological materials

The set of *Triticum aestivum* Chinese Spring deletion and nullisomic-tetrasomic lines for chromosome 1 and 6 were obtained from the Wheat Genetics Resource Center (WGRC, Kansas State University, Manhattan, KS). Except for *Triticum aestivum* cv. Summit and Bobwhite, the wheat varieties used were obtained from the U.S. National Genetic Resource Program grain repository. *Triticum aestivum* cv. Bobwhite was obtained from Dr. Ann Blechl at USDA and *Triticum aestivum* cv Summit was obtained from its developer Dr. Robert Machett (deceased) while he was at Resource Seeds Inc. (now part of ChemChina).

## Procedure

### Gel casting

Ingredients for various gel concentrations can be found in Table 1. The recipe makes two gels; 12% acrylamide concentration is best for resolving gliadins, and is the default through this paper.

1. Combine all ingredients from the table except the catalyst TEMED in a 125 mL side-arm flask. Add acrylamide in a fume hood. Swirl to mix until urea is completely dissolved.
2. De-gas the solution. Cover the top of the flask with a large rubber stopper and attach vacuum hose to side-arm of flask. Apply vacuum to the flask and swirl it gently to remove bubbles if they appear. Leave under vacuum for 30 min.
3. Turn off the vacuum and remove the stopper. Add TEMED in a fume hood. Swirl to mix.
4. Carefully pour or pipette gel solution into an empty cassette until completely full.
5. Insert the comb by starting at one end at an angle, then slowly lower it down in place to avoid air bubble formation. If necessary, top off the cassette with gel solution. Save the excess gel solution until your gels are set.
6. Place damp paper towels on top of the cassettes before placing inside an incubator to prevent the solution at the top of the cassette from drying.
7. Incubate cassettes at 37 °C for 1 h to allow the gel to polymerize faster.
8. Pre-run solidified gels before use.

**Table 1:** A-PAGE reagents.

Reagents for 20 mL solution (good for 2.0 gels)					
	Final Concentration	8% gel	10% gel	12% gel	15% gel
**Urea**	5 M	6 g	6 g	6 g	6 g
Water (ultrapure)*	–	10 mL	9 mL	8 mL	6 mL
**Acetic Acid (50%)**	5%	2 mL	2 mL	2 mL	2 mL
**10% APS**	0.2%	0.4 mL	0.4 mL	0.4 mL	0.4 mL
**Tris pH 8.5 (1** **M)**	12.5 mM pH 3.1	0.25 mL	0.25 mL	0.25 mL	0.25 mL
**Acrylamide (40%)**	8/10/12/15%	4 mL	5 mL	6 mL	7.5 mL
**TEMED®**	1%	200 µL	200 µL	200 µL	200 µL

⁎ Add water to a final volume of 20 ml.

### Gel pre-run

**Fig. 1:**
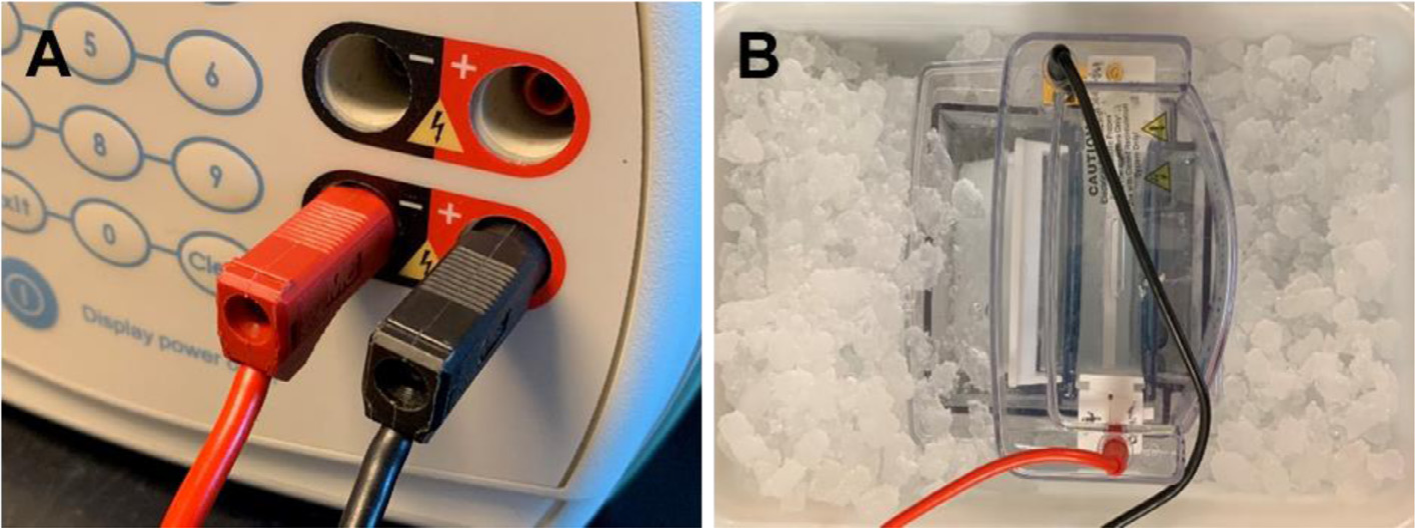
A-PAGE running conditions. (A) A-PAGE is run on reverse polarity. This is accomplished by connecting the gel rig anode to the negative pole and the cathode to the positive pole of the power supply. (B) A-PAGE is run under cold conditions. Thin gels (1 mm) can overheat and melt when run at high voltage. This risk is mitigated by placing the gel rig on an ice-cold water bath.

A-PAGE is run on **reverse polarity**. The electrodes are connected opposite to how they are in SDS-PAGE. The electrodes of the SureLock™ Mini-Cell are connected to a power supply with the cathode (−) at the bottom of the gel and the anode (+) at the top. Proteins in A-PAGE gels are highly protonated by the low-pH environment, which causes them to migrate from the anode (+) toward the cathode (−). On the Mini-Cell, the red electrode should be connected to the black power source, and vice-versa (Fig. 1, panel A).

Pre-running the gel removes impurities, and results in sharper protein bands.

1. Remove the comb and the white sticker covering the slot on the back of the cassettes. The gel comb is reusable, simply wash in deionized water and dry for future use.
2. Place the gel cassettes in a Mini-Cell chamber with the shorter “well” side facing the inside, then lock them in place with the gel tension wedge.
3. Fill the inner and outer chamber of the Mini-Cell with 5% acetic acid as running buffer. The inner chamber should be full but not overflowing. The outer chamber should be filled to about 3 cm from the top of the Mini-Cell.
4. Clean out the wells by gently pipetting buffer into them without disturbing the well sample separators. You should see their edges become more clearly defined.
5. Pre-electrophorese at room temperature for 90 min at 150 V, or until mA reading has stabilized. Make sure that the pre-run is on reverse polarity.
6. After the pre-run, gels can be used immediately. Note: you can save the running buffer for future runs and pre-runs.
7. If pre-run gels will not be used immediately, store gels at 4 °C wrapped in plastic to avoid desiccation. Note that we got the best results if we ran gels the same day that we cast them.

### Gel run

1. Resuspend dry protein samples in equal parts 5% acetic acid and A-PAGE sample solution (4.5 M urea in 5% acetic acid with a few grains of methyl green). If samples have already been resuspended and are coming out of storage at 4 °C, warm them at 70 °C for 5 min, then vortex and briefly spin down before loading.
2. Label the outside of each gel cassette to appropriately identify them.
3. Load two gel cassettes or one cassette and one blank (buffer dam) into a Mini-Cell, seal tight by locking the gel tension wedge, and add buffer. Fill the upper and lower chamber with 5% acetic running buffer. Fill outer chamber to about 3.0 cm below the top of the gel.
4. Clean the wells before loading samples by gently pipetting buffer 3–5 times into each well.
5. Load 5–20 µL sample in each well (depending on protein concentration).
6. Place Mini-Cells in a plastic tray and fill tray with ice. Add liquid water to make an ice bath to improve heat transfer (Fig. 1, panel B).
7. Run gels following the recommended time and power shown in Table 2. **Remember to run gels with reversed polarity**.

**Table 2:** A-PAGE running conditions.

Running Times by Concentration and Voltage								
Concentration	8% acrylamide		10% acrylamide		12% acrylamide		15% acrylamide	
Voltage	250V	400V	250V	400V	250V	400V	400V	500V
Base Run Time	90 min	50 min	210 min	90 min	270 min	100 min	120 min	110 min

Modifiers: • If running only one gel per Mini-Cell, run for 10% more time. • If casting and running on the same day, run for 20% more time.

### Gel staining and de-staining

1. Shake the Coomassie stain solution well before use, then add 50 mL to a gel staining box.
2. Remove the gel from its cassette by prying the cassette apart with a metal spatula. Place one gel into the staining box.
3. Put a lid on the staining box and stain gel at room temperature with shaking (about 50 rpm). Stain gels overnight for convenience, however, staining for as little as 4 h is possible.
4. When protein bands are stained, dispose of Coomassie gel stain solution in accordance with local guidelines. (Methanol is hazardous, and the solution will be approximately pH 1.8.)
5. Rinse the gel 2–3 times in water, then de-stain in water for 15 min with shaking by placing the staining box in an orbital shaker. Repeat this process until the desired level of protein staining is obtained.

### Gel imaging

Use a camera or a document scanner to image gels. If you are using a document scanner, it is best to cut off the raised area at the bottom of the gel to obtain a flat surface to scan.

### Sample preparation

There are several methods already published on the fractionation of glutenins and gliadin proteins from flour [19,20]. In this report, the glutenin and gliadin protein fractions were separated using 70% ethanol. For phenotyping selected *T. aestivum* Chinese Spring deletion lines and wheat varieties, about 10 mg of milled flour was used as input. For screening of mutant lines, flour from the crushed brush-end of half-seed was used. The embryo-end of the half-seeds of identified mutants were planted for bulking and further characterization.

## Results and discussion

A-PAGE is commonly used to identify wheat varieties based on their gliadin protein profiles. The most frequently used method is the one developed by Bushuk and Zillman in 1977 [21] with the lactate-lactic acid buffer system first described by Jones in 1959 [4]. The Bushuk and Zillman A-PAGE method was further modified by Khan et a1. in 1983 [22] and by Metakovsky and Novoselskaya in 1991 [23]. Metakovsky and Novoselskaya described in their 1991 paper the observed “day-to-day experimental variation in gliadin spectrum” despite the use of a standard procedure performed by a technically skilled and experienced operator. An acetic acid-based A-PAGE method was described by Clements in 1988 [24] that successfully separated wheat gliadins with better resolution and sensitivity compared to the lactate-lactic acid based method. In 1994, Morel modified Clements’ A-PAGE to resolve wheat glutenins by adding urea and adjusting the pH of the gel composition to pH 3.1, similar to the lactate-lactic acid method [25].

Here we describe the use of an acetic acid based A-PAGE to separate gliadins in wheat. We adapted an A-PAGE method with urea that is routinely used in the purification and characterization of microbial antibiotic peptides [1]. This denaturing A-PAGE separates proteins based on size and charge. This denaturing condition removes the conformational variability that contributes to additional protein polymorphism in lactate-lactic acid based A-PAGE gel gliadin profiles. The original method from Porter et al. was modified by adjusting the gel pH to 3.1, which was deemed to be optimal for gliadin solubility. We further modified the protocol by using commercially available reagents and materials to simplify and make gel casting more efficient. The use of commercially available empty gel cassettes prevented leakage problems during gel casting and saved time.

### Method validation

*Triticum aestivum* Chinese Spring has been the standard reference in hexaploid wheat research to compare and improve inter-laboratory experimental results. Therefore, we validated our new A-PAGE method by comparing the gliadin profiles of a set of Chinese Spring deletion and aneuploid lines that are missing genomic regions that encode for gliadins.

**Fig. 2:**
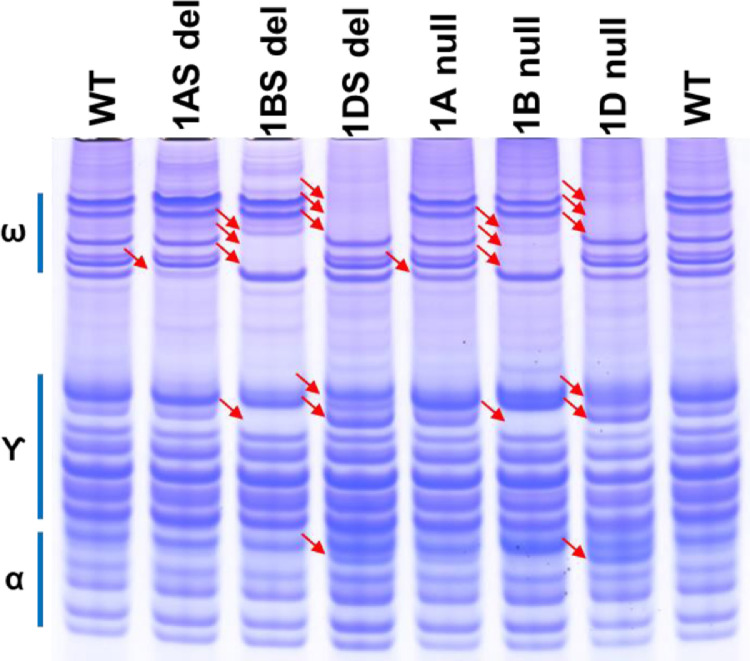
A-PAGE gliadin profiles of Chinese Spring chromosome 1 deletion lines. Lanes 1 and 8, WT is progenitor Chinese Spring; lanes 2–4, chromosome 1 short arm deletion lines WGRC 4510-L1, 4512-L1 and 4514-L3; lanes 5–7, chromosome 1 nullisomic-tetrasomic lines WGRC 3257 (N1A-T1B), 3259 (N1B-T1A), and 3262 (N1D-T1B). The label on the right indicates the position of the omega, gamma and alpha gliadins on the gel. The red arrows indicate the positions of altered proteins, either new or missing or with reduced or elevated level.

As shown in Fig. 2, the electrophoretic profile of deletion lines without the *Gli-A1* (1AS del), *Gli-B1* (1BS del), and *Gli-D1* (1DS del) loci in the short arm of chromosome 1 have the same omega- and gamma-gliadin bands missing or with altered intensities as in corresponding nullisomic-tetrasomic lines that are missing the whole chromosome 1A, chromosome 1B or chromosome 1D. The deletion in 1AS showed a faint band missing in the lower omega-gliadin region, the same as in 1A nullisomic. The 1BS deletion and the 1B nullisomic has the same 3 omega-gliadin bands missing, a more intense omega-gliadin band and a missing gamma-gliadin band. The 1DS deletion and 1D nullisomic are both missing omega-gliadin 1,2 bands and one missing and one new gamma-gliadin protein bands. The positions of the omega- and gamma-gliadins generated by this new A-PAGE protocol is consistent with the gel positions of similar gliadins in previously published works using the canonical lactate-lactic acid based A-PAGE gels.

**Fig. 3:**
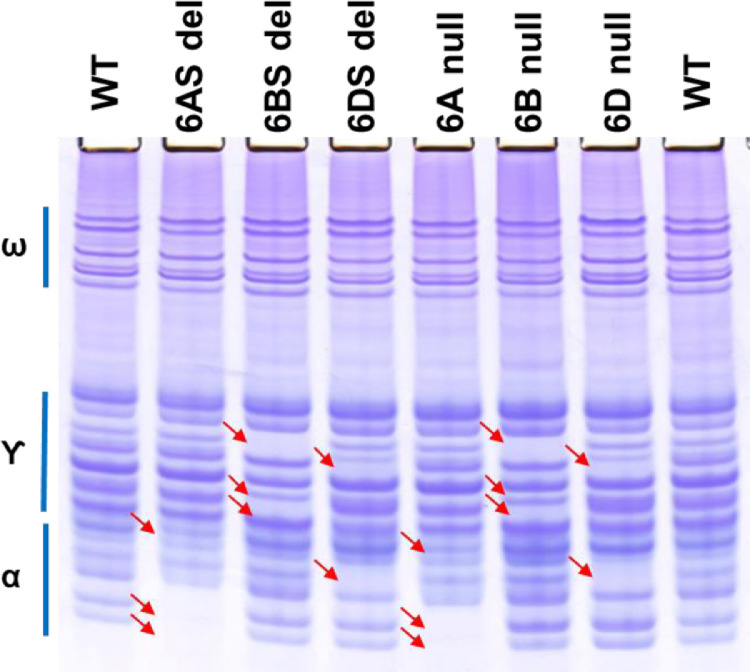
A-PAGE seed protein profiles of Chinese Spring chromosome 6 deletion lines. Lanes 1 and 8, WT is progenitor Chinese Spring; lanes 2–4, chromosome 6 short arm deletion lines WGRC 4540-L5, 4542-L1, and 4544-L2; lanes 5–7, chromosome 6A nullisomic-tetrasomic lines WGRC 3152 (N6A-T6B), 3154 (N6B-T6A), and 3156 (N6D-T6B). The label on the left indicates the position of the omega-, gamma- and alpha-gliadins on the gel. The red arrows indicate the positions of altered proteins, either new or missing or with reduced or elevated level.

Similarly, the deletion of the loci coding for alpha-gliadins in the chromosome 6 group, *Gli-A2, Gli-B2*, and *Gli-D2* exhibited similar patterns of missing alpha- gliadins as in the nullisomic-tetrasomic lines for chromosome 6 (Fig. 3). The 6AS deletion (WGRC 4540-L5) and 6A nullisomic (WGRC 3152) showed two missing lower alpha-gliadin and a missing band in the upper alpha-gliadin region (revealing a fainter band behind it). The deletion in *Gli-B2* on 6BS (WGRC 4542-L1) and in the corresponding nullisomic for chromosome 6B showed three missing protein bands all of which are in the gamma-gliadin region. Chromosome 6D nullisomic and WGRC 4544-L2 *Gli-D2* deletion line have two missing protein bands compared to wild type – one in the alpha-gliadin region and another in the gamma-gliadin region. Are there gamma-gliadin like proteins in the short arm of chromosome 6 group? Do these alpha-gliadin proteins have more positively charged residues at lower pH thus migrate slower? It will be of great interest to identify and further characterize the proteins that migrated in the gamma-gliadin region that mapped to the *Gli-B2* and *Gli-D2* loci.

### Tool to phenotype wheat varieties

The wide array of polymorphisms at the gliadin loci serves as good genetic markers to track wheat varieties’ phenotypes [26]. Although genomic tools such as the wheat 90 K SNP array [27] are now available to genotype wheat varieties at a higher resolution, the use of A-PAGE method still offers a fast and inexpensive way to verify the phenotype or purity of wheat varieties especially for smaller breeding programs.

**Fig. 4:**
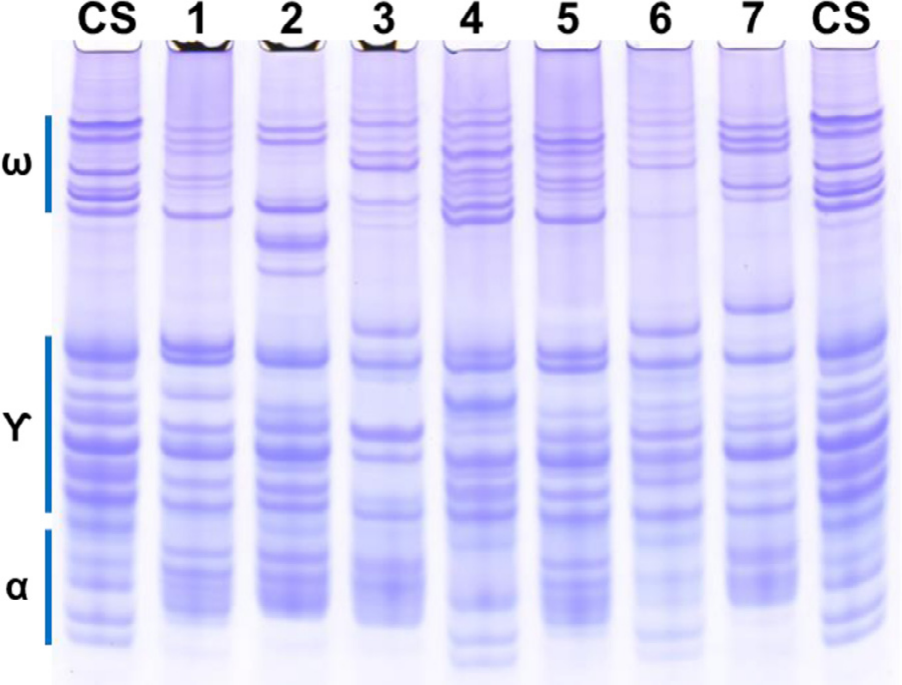
A-PAGE gliadin profiles of flour protein from different wheat varieties. A-PAGE can be used to phenotype different wheat varieties. Shown are the gliadin profiles of different *Triticum aestivum* varieties relative to Chinese Spring (CS; NSGC Citr 14108 TR09ID SD); lane 1, Summit; lane 2, Bobwhite; lane 3, Cheyenne (NSGC Citr 8885 TR04ID SD); lane 4, Thatcher (NSGC Citr 10003 TR98ID SD); lane 5, Yecora rojo (NSGC Citr 17414 TR04ID SD); lane 6, Norstar (NSGC Citr 17735 TR94ID SD); lane 7, Norin 61 (NSGC PI 182591 TR97ID SD).

As shown in Fig. 4, the gliadin profiles for eight common wheat cultivars resolved more than 20 distinct protein bands per line. Each cultivar exhibited a unique gliadin profile. All three classes of gliadins (omega, gamma and alpha) showed high protein band polymorphism between cultivars. The gliadin protein bands not only differed in position on the gel but also in number and intensity. For example, Thatcher exhibited at least 9 bands in the omega-gliadin region, whereas, Summit and Norin 61 have only 6; Thatcher shared two of the lower alpha-gliadin bands only with Chinese Spring and Norstar.

### Screen for wheat gliadin mutants

Our primary intention in developing this modified A-PAGE protocol was to screen a mutagenized population of a commercial bread wheat variety (*T. aestivum* cv Summit) for mutations in the gliadin encoding loci in chromosome groups 1S and 6S. Our criteria which included easy to cast and sturdy gels, short run time and consistent protein resolution were achieved with the new protocol. The ease in casting gels and shorter run time allowed two summer undergraduate students to screen 1139 mutant lines (3 to 4 seeds assayed per line) in just eight weeks.

**Fig. 5:**
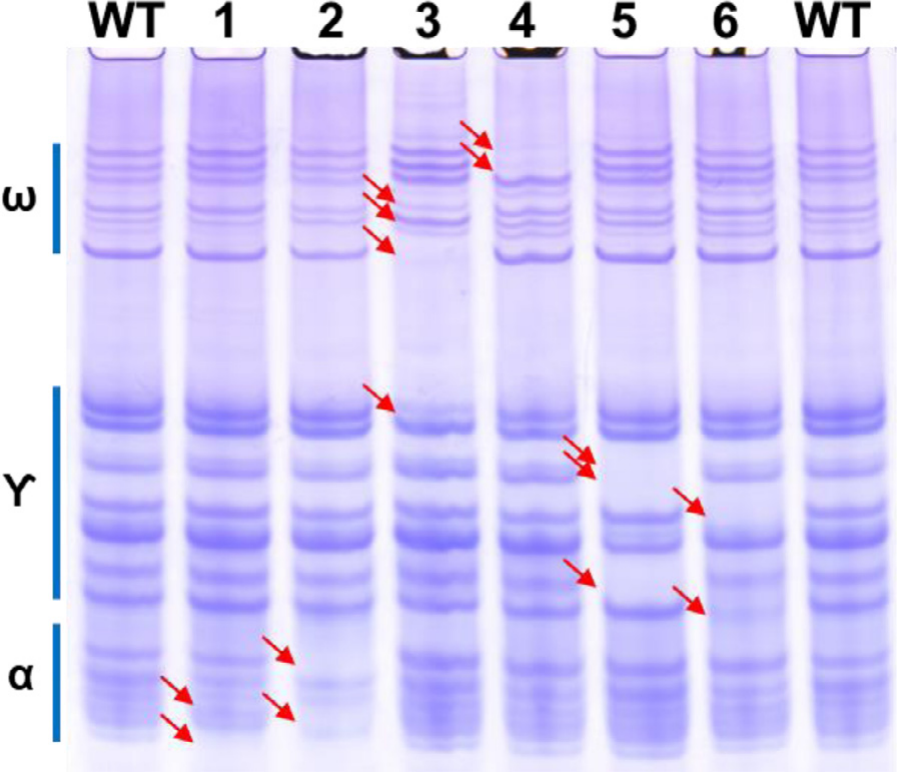
A-PAGE to screen for gliadin mutant lines. A-PAGE gliadin profiles of different mutants identified by screening a fast-neutron radiation mutagenized population of *Triticum aestivum* cv. Summit, a high-yielding commercial bread wheat variety. WT is the progenitor Summit; each of the following six lanes shows the protein profiles from independent mutant lines deficient in different gliadins. Lanes 1–2, alpha gliadin deficient lines F103B and F212B; lanes 3–4, omega and gamma gliadin deficient lines F255D and F77A; lanes 5–6, gamma gliadin deficient lines F94D and F204F. Red arrow indicates the position of missing or new gliadin bands.

We identified 36 candidate mutant lines with 12 each that are deficient in omega-, gamma- and alpha-gliadins. Two independent deficient line per gliadin type are shown in Fig. 5. Compared to the protein profile of the wild type Summit progenitor, the absence of bands or presence of new gliadin bands in the mutant lines are clear and distinct. We also used the new A-PAGE protocol to track and verify the mutations during the backcrossing process to reduce the unintended mutations in the background.

### Native A-PAGE

**Fig. 6:**
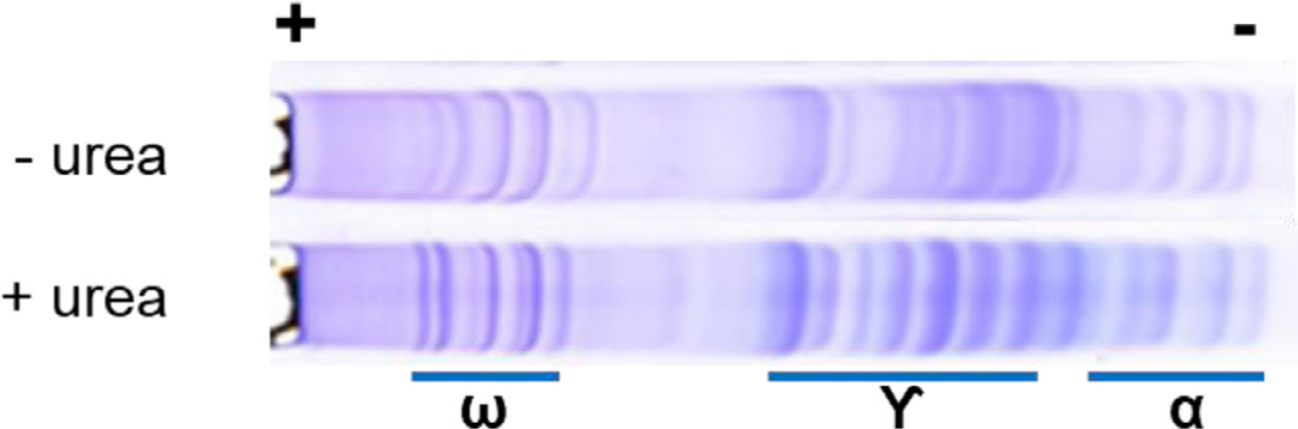
Native A-PAGE. A-PAGE gliadin profiles of wild type *T. aestivum* Chinese Spring with (denatured) or without urea (non-denatured). The protein migrated from the anode (+) to the cathode (−) end of the gel as indicated at the top. The labels at the bottom indicate the position of the omega, gamma and alpha gliadins on the gel.

The A-PAGE gel composition in this protocol can be further modified to make it a non-denaturing gel by removing urea. The urea in the sample solution can be replaced by 5% glycerol. As shown in Fig. 6, the protein profile of the Chinese Spring gliadins resolved with or without urea exhibited distinct differences. The protein bands at the gamma-gliadin region are the most highly altered in terms of number, position and intensities. The protein bands appear to be more distinct in A-PAGE with urea. It is possible that the shape of proteins in a non-denaturing condition could be in flux between intermediate conformations prior to entering the gel hence the tendency to exhibit a few more “fuzzy” bands. Researchers can use this new method with either denaturing or non-denaturing gel composition using the same protocol depending on the goal of the experiment. Note, however, that in our hands A-PAGE gels cast without urea tend to be weaker and needed to be incubated at 37 °C longer to solidify.

### Additional information

1. The 5% acetic acid running buffer can be re-used at least four times.
2. Gels can be re-stained if bands are too light due to excessive destaining.
3. A detailed instruction on the use and care of the XCell SureLock™ Mini-Cell equipment used in this report can be found at the company's website (https://www.thermofisher.com/order/catalog/product/EI0001)
4. The commercially available empty cassette used in this protocol was made to fit XCell SureLock™ Mini-Cell but any other devices where it is compatible should be usable.
5. Commercially available Coomassie Blue stains can be used to detect the protein bands in an acid-PAGE gel. For homemade stain we use the Kasarda Coomassie Blue stain recipe as follows: Dissolve 0.6 g of Coomassie Brilliant Blue R in 125 ml of methanol. Add 400 ml deionized water. Add 50 g ammonium sulfate, 5.8 ml of 85% phosphoric acid (do in the fume hood) and deionized water to raise the volume to 625 ml. Mix well. This stain can be stored at room temperature until used.
